# Chronic Skin Disease, Media Use and Health Values in the Quality of Life of Adolescents

**DOI:** 10.3390/children13070899

**Published:** 2026-07-06

**Authors:** Katalin Julianna Dinnyés, Zsanett Renáta Csoma

**Affiliations:** 1 Health Behaviour and Development Group, Faculty of Health Sciences and Social Training, University of Szeged, H-6726 Szeged, Hungary; csoma.zsanett@med.u-szeged.hu; 2 Doctoral School of Education and Society, University of Pécs, H-7622 Pécs, Hungary; 3 Department of Dermatology and Allergology, Albert Szent-Györgyi Clinical Center, University of Szeged, H-6720 Szeged, Hungary

**Keywords:** adolescents, dermatology, quality of life, social media use, screen time, body image, self-esteem, health values, psychodermatology

## Abstract

**Highlights:**

**What are the main findings?**

- Greater dermatology-related quality of life impairment was associated with poorer quality of life, lower self-esteem, more negative body image, and higher anxiety levels among adolescents with chronic dermatological diseases.
- Higher screen time is linked to worse dermatological quality of life, while stronger health values are associated with more favourable health behaviours and better well-being.

**What are the implications of the main findings?**

- Psychosocial factors (self-esteem, anxiety, body image) should be routinely integrated into adolescent dermatological care.
- Health promotion programs should focus on media literacy and strengthening health values to mitigate the negative effects of social media on adolescents.

**Abstract:**

Introduction: Chronic dermatological diseases that appear in adolescence, such as acne vulgaris, atopic dermatitis, alopecia areata and psoriasis vulgaris, not only cause physical symptoms but also significantly affect young people’s quality of life, mental state, self-esteem and social relationships. Social media, especially information spread by influencers, significantly influences adolescents’ body image, health-related attitudes and even the quality of the physician-patient relationship. The aim of our study was to explore the relationships between dermatology-related quality of life, media use, health values, body image and self-esteem among adolescents with chronic dermatological diseases. Methods: In our cross-sectional, quantitative study, we used validated questionnaires (DLQI, EQ-5D-5L, IRVS, Attitude Scale, STAI-Y2, SWLS-H, Rosenberg Scale, BAT), which we supplemented with a media consumption questionnaire of our own design. Structured data collection took place between October 2024 and March 2025, with the participation of 208 adolescents aged 11–18. Data analysis was performed using SPSS 26.0 (Spearman’s correlation, Mann–Whitney test). Differences were considered significant at *p* < 0.05. Ethical approval: BM/22429-1/2024. Results: Acne vulgaris was the most common diagnosis (65%), followed by atopic dermatitis (22%) and psoriasis (11%). Over a quarter of the adolescents (27%) followed influencers who provided skincare advice. The mean daily screen time was 4.5 h, with 3.7 h on smartphones. A longer screen time was significantly correlated with poorer dermatological quality of life. Greater dermatology-related quality of life impairment (higher DLQI scores) was associated with poorer general quality of life (EQ-5D-5L). Following skincare-related influencers was significantly associated with dermatology-related quality of life and anxiety. Conversely, stronger health values were significantly linked to more favourable health behaviors. Conclusions: In this sample, greater dermatology-related quality-of-life impairment was associated with poorer psychosocial outcomes. Longer screen time was associated with poorer dermatology-related quality of life and less favourable psychosocial outcomes. The novelty of our study lies in the use of a self-developed media consumption questionnaire, which is suitable for the complex mapping of psychological and quality-of-life factors in adolescents.

## 1. Introduction

Adolescence is a period of physical, psychological and social change, during which young people gradually seek their identity and independence. At this stage of development, peer relationships and community experiences become more important, while the role of parents fades into the background. The family, especially parents’ attitudes and behaviours, continues to have a significant influence on adolescents’ health behaviours and psychosocial well-being. This stage of life can be further complicated by chronic dermatological diseases, which are associated with visible symptoms and long-term treatment, posing challenges not only on a physical level but also on a psychological and social level [1,2,3]. The most common chronic dermatological conditions in adolescence include acne vulgaris, atopic dermatitis, alopecia areata and psoriasis vulgaris. These diseases can significantly impair quality of life: in many cases, the symptoms are conspicuous and easily noticeable to others, leading to stigmatisation and exclusion. International research confirms that the lifestyles and subjective health assessments of young people with chronic allergic or dermatological conditions differ significantly from those of their healthy peers, particularly in terms of physical activity and health-conscious behaviour [4]. Further studies have confirmed that skin problems are often associated with lethargy, depression, anxiety and a negative body image. These factors are particularly prevalent among adolescents, as physical appearance and social acceptance are of paramount importance at this stage of development. Stress is a key factor in the development and persistence of chronic skin diseases. Gieler and colleagues emphasised that psychological stress and social stressors can trigger flare-ups of symptoms [5]. According to Német and colleagues, multidisciplinary care combining dermatological treatment with psychological support can significantly improve quality of life [2,3]. This is particularly important in adolescents, in whom chronic illness can cause not only physical complaints but also identity and self-esteem problems. In their work, they describe how participants in the study often experience a deterioration in quality of life and psychosocial stress, with frequent accompanying psychological disorders in cases of chronic dermatological disease.

The foundations of health awareness in adolescence are decisive for health behaviour in adulthood. The literature emphasises that developing appropriate lifestyle habits early on is much easier and more effective than correcting them later in life [6]. Values, including health, act as motivating forces, influencing decision-making and everyday behaviour [7,8]. According to Meleg, our lives are characterised by a series of decisions determined by our values [9]. It is therefore important to examine the role of health in adolescents’ value systems, which determine their attitudes towards prevention, healthy lifestyles, and diseases. Among young people with skin problems, health as a value is particularly important, as experiencing the disease often causes them to reevaluate their attitudes towards health. The value dimension of health is closely linked to health-conscious behaviour, including dietary habits, sports activities, and therapeutic adherence. In recent years, the media, especially social media, has become a defining factor in adolescents’ lives. According to a 2023 survey, 95% of 13- to 17-year-olds are active social media users [10]. Hungarian adolescents spend an average of 5–8 h a day in front of a screen, whereas in the United States, screen time reaches 7 h a day [6]. Excessive media use is closely linked to depression, anxiety and behavioural problems. Social media content has a strong opinion-forming effect and is shaped by online content [11,12]. Panjrath and Tiwari emphasise that unrealistic beauty ideals and content conveyed by influencers cause dissatisfaction, anxiety and self-esteem issues [13].

The media can also influence the physician-patient relationship, as patients often base their decisions on information from online forums, which can potentially lead to therapeutic nonadherence [14]. Young people’s body image is thus shaped not only by their own experiences but also by the norms conveyed by online content, which increases the risk of body image disorders and self-esteem problems [11]. One of the most important determinants of adolescents’ health behaviour and psychological well-being is the quality of the parent–child relationship. Parents’ attitudes towards health, media use and parenting style can directly influence their children’s self-esteem and health behaviour [15]. Szabadi, Kis and Józsa also confirmed that the emotional atmosphere of parental treatment, whether supportive or restrictive, has a strong effect on children’s emotional and social development [16,17]. In their work, Obál and colleagues reported that parental parenting style is related to adolescents’ psychological state and health behaviour [18]. Dominantly demanding parental behaviour reduces adolescents’ self-esteem, whereas a warm, supportive atmosphere strengthens their self-confidence and autonomy. According to Li et al., who confirmed the findings of Drjenovszky and Sztáray Kézdy, the emergence of the modern father role also positively affects family cohesion and the psychological development of children [19,20]. Adolescents’ therapeutic cooperation in the management of chronic dermatological diseases can be strongly influenced by their parents’ attitudes. If parents are critical and verify information sources, it can strengthen therapy adherence, whereas superficial information from social media can easily lead to uncertainty and nonadherence [21]. On the basis of our review of the literature, chronic dermatological diseases, psychosocial factors, media use and parental attitudes clearly interact in complex ways to shape adolescents’ quality of life, body image and self-esteem. The physical and psychological burdens of chronic illness, unrealistic media influences and the quality of parental support all determine the extent to which adolescents are able to value their health and make health-conscious decisions. Our work is interdisciplinary, combining findings from psychology, dermatology, health sociology, media research, and education science. A holistic approach not only contributes to scientific cooperation but can also support the development of practical prevention and health promotion programs.

### 1.1. Study Aim

This cross-sectional study aimed to examine the quality of life related to dermatology and its associations with general quality of life, self-esteem, body image, anxiety, health behaviour, health-related values and media use among adolescents with chronic dermatological diseases.

The secondary objective was to describe the digital media habits of this clinical adolescent sample, including screen time, social media use, consumption of short-form videos, and following skincare-related influencers.

Exploratory analyses were conducted to examine the associations between screen time, social media use, and health-related values, and psychosocial and health-behaviour indicators. Due to the cross-sectional design, the study aimed to identify associations rather than causal effects, mediation or moderation pathways.

### 1.2. Hypotheses

**H1.**  *Greater dermatology-related quality of life impairment (DLQI) is associated with lower self-esteem, poorer quality of life and higher anxiety*.

**H2.**  *Higher media use and screen time are associated with a more negative body image, lower self-esteem, and increased anxiety*.

**H3.**  *A stronger internalisation of health values is positively associated with better health behaviour, higher life satisfaction, and a better quality of life*.

## 2. Materials and Methods

### 2.1. Study Design

This cross-sectional quantitative study used a self-administered questionnaire to collect personal and psychosocial data. The study adhered to the STROBE recommendations for cross-sectional studies regarding the sampling frame, inclusion/exclusion criteria, and outcome/variable definitions. Participation was voluntary and anonymous, and adolescents were informed that they could withdraw at any time without consequence.

### 2.2. Participants

The target population comprised adolescents diagnosed with chronic dermatological diseases. A total of 208 individuals (102 females (49%) and 106 males (51%) participated in the study. The mean age of participants was 15.3 years (SD = 2.3), mean body weight of 63.07 kg, and mean height of 1.68 m.

The most common disease was acne vulgaris (N = 127, 61.1%), followed by atopic dermatitis (N = 38, 18.3%) and psoriasis (N = 22, 10.6%). Less common conditions included alopecia areata (N = 2, 1.0%), acne vulgaris and atopic dermatitis (N = 8, 3.8%), acne vulgaris and seborrhea capitis (N = 2, 1.0%), acne vulgaris and alopecia areata (N = 2, 1.0%), psoriasis and atopic dermatitis (N= 2, 1.0%), and acne vulgaris and psoriasis (N = 2, 1.0%). The average body weight of the participants was 63.07 kg, and the average height was 1.68 m.

### 2.3. Inclusion Criteria

- Adolescents aged 11–18 years.
- A confirmed diagnosis of a chronic dermatological condition (e.g., acne vulgaris, psoriasis, atopic dermatitis or alopecia areata) by a dermatologist.
- Participation with informed consent (and parental consent if under 18).

### 2.4. Exclusion Criteria

- Presence of other chronic somatic or psychiatric conditions that influence quality of life or self-esteem.
- Incomplete questionnaire data (excluded via listwise deletion).
- Multiple submissions (only the most complete entry is retained).

### 2.5. Recruitment and Setting

Participants were recruited between September 2024 and March 2025 at the Paediatric Dermatology Outpatient Clinic of the Department of Dermatology and Allergology at the Albert Szent-Györgyi Clinical Centre, University of Szeged. Recruitment occurred during routine dermatological visits. Adolescents and their parents/carers received written and verbal information, and only those who provided informed consent participated.

A priori power analysis (G*Power v3.1) indicated that, to detect a small-to-medium effect size (r = 0.20) with 95% power and α = 0.05, a minimum sample size of 193 would be required. Thus, the final sample size of 208 ensured adequate statistical power.

### 2.6. Measuring Instruments

The questionnaire comprised 74 items, combining validated instruments and self-developed media-habit questions. All instruments were available in a validated Hungarian version.

- Sociodemographic variables included age, gender, weight, height, residence and family background.
- The Dermatology Life Quality Index (DLQI) measures the impact of skin disease on daily activities, symptoms, emotions and social relationships (10 items, 4-point Likert scale; higher scores indicate a worse dermatological quality of life) [22]. Scores of 0–1 indicate no effect on the patient’s life, 2–5 a small effect, 6–10 a moderate effect, 11–20 a very large effect, and 21–30 an extremely large effect.
- The EQ-5D-5L general health-related quality of life was assessed using the EuroQol EQ-5D-5L questionnaire, which evaluates five dimensions (mobility, self-care, usual activities, pain/discomfort and anxiety/depression), each of which is rated on a scale of five levels of severity. The questionnaire also includes a visual analogue scale (EQ VAS; 0–100), where higher scores indicate a better perceived health status [23].
- The Rosenberg Self-Esteem Scale (RSES) consists of 10 items rated on a four-point Likert scale (1 = strongly disagree to 4 = strongly agree). Negatively worded items were reverse-coded before calculating the total score. Total scores range from 10 to 40, with higher scores indicating higher self-esteem [24].
- The State-Trait Anxiety Inventory (STAI-Y2) comprises 20 items assessing stable anxiety traits on a 4-point scale, with higher scores reflecting greater anxiety [24].
- The Body Attitudes Test (BAT) and the Body Shape Questionnaire (BSQ-14) evaluate body image, dissatisfaction and preoccupation with appearance; higher scores indicate a more negative body image [24].
- Health Attitude and Behaviour Scale: Consists of 35 items assessing ten domains (nutrition, physical activity, prevention, smoking, alcohol, substance use, aggression, internet use, subjective health and emotional balance). Higher scores indicate more favourable health behaviour [25].
- The Hofmeister-Tóth & Neulinger Value Scale measures the importance and realisation of health-related values on a 5-point Likert scale. Higher scores indicate a stronger internalisation of health as a value [25].
- This study used a self-developed media use questionnaire to explore the digital media habits of adolescents with chronic dermatological diseases. It included questions about digital device ownership and usage, daily screen time, smartphone screen time, preferred social media platforms, app usage, internet usage, short-form video consumption (e.g., TikTok, Reels, and YouTube Shorts), following influencers, media-related health information, and the perceived influence of social media on body image, health behaviors, and skincare decisions. The media use questions were developed specifically for the present study and were used as an exploratory, non-validated set of items to describe adolescents’ digital media habits.

### 2.7. Reliability

Internal consistency was verified using Cronbach’s alpha for each scale: DLQI (α = 0.561; moderate); EQ-5D-5L VAS (α = 0.984; excellent); RSES (α = 0.870; excellent); STAI-Y2 (α = 0.830; high); BAT (α = 0.847; good); BSQ-14 (α = 0.740; acceptable); and Health Attitude Scale (α = 0.839; good). These results demonstrate that the reliability of all instruments is adequate to excellent.

### 2.8. Statistical Analysis

The data were processed using IBM SPSS Statistics version 27 and Microsoft Excel version 2010. The analyses included:

- Descriptive statistics (means, standard deviations, frequencies and percentages),
- Normality tests (Kolmogorov–Smirnov), which confirmed non-normal distribution (*p* < 0.05).
- Nonparametric analyses due to ordinal scaling and non-normality.
- Mann–Whitney U and Kruskal–Wallis H tests for group comparisons.
- Spearman’s rank correlation to examine associations between variables.

Effect sizes (r and η^2^) and exact *p*-values were reported in accordance with APA guidelines. Missing data (<5%) were excluded by listwise deletion. The significance level was set at *p* < 0.05 (two-tailed).

### 2.9. Ethical Considerations

The study was approved by the Scientific and Research Ethics Committee of the Health Sciences Council, Hungary (Approval No. BM/22429-1/2024). All procedures complied with the Declaration of Helsinki. Written informed consent was obtained from adolescents and their parents (for participants under 18). Participation was anonymous, and data protection adhered to GDPR standards; no direct identifiers were collected.

### 2.10. Data Access

The datasets generated and analysed during the current study are available from the corresponding author on reasonable request. Data are stored in a de-identified format, accompanied by a variable codebook and scoring guide for replication and secondary analysis.

## 3. Results

In the study sample, the average score on the Dermatology Life Quality Index (DLQI) was 2.81 (due to the scale’s consolidation, the minimum = 0, maximum = 30). When placed within the scale’s interpretation range, this value indicates that, on average, chronic dermatological diseases have a small impact on adolescents’ lives.

The body image attitudes of the participating adolescents were measured via the Body Attitudes Test (BAT). The overall average score was 25.53 (minimum = 0, maximum = 100, due to the consolidation of the validated measurement tool’s results). This result shows that adolescents have moderate body image problems. The average value falls within the lower-middle range of the scale, suggesting that some young people struggle with critical self-assessment, are dissatisfied with their bodies, or are anxious about their appearance, but that severely distorted body image is not typical of the sample as a whole. We also examined adolescents’ dissatisfaction with their body image via the BSQ-14 questionnaire. The overall average score was 29.06 (minimum = 0, maximum = 80 due to scale consolidation), indicating that the study sample’s average score did not reach the clinical threshold for body image disorders. These findings suggest that most adolescents do not exhibit severe body image disturbance. In the study sample, the majority of the adolescents had positive self-images and stable self-esteem, although there may be individual differences, with lower scores indicating more uncertain self-esteem. The results suggest that the self-esteem of adolescents with chronic dermatological conditions does not show any serious deviations overall, but that self-esteem may be closely related to body image, appearance and social relationships. Adolescents’ satisfaction with life was measured using the SWLS-H questionnaire. The overall average score was 26.8 (minimum = 5, maximum = 30 due to the consolidation of the measurement tool results), which falls within the upper range of the scale, indicating that the majority of adolescents are fundamentally satisfied with their lives. We examined the level of anxiety via the STAI-Y2 questionnaire. The average score was 39.1 (minimum = 20, maximum = 60 due to the consolidation of the scale), which indicates an elevated level of anxiety among adolescents. Psychological state and anxiety may be closely related to health behaviour, so we examined attitudes towards health.

**Figure 1:**
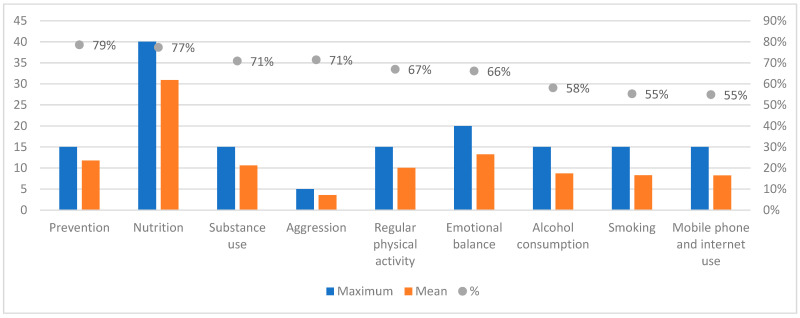
Adolescents’ health behaviour.

We examined adolescents’ attitudes towards health in several dimensions. The results are shown in Figure 1. In this case, the higher the value obtained, the more favourable the respondent’s health behaviour is in that dimension.

As shown in Figure 1, the highest values were observed in the areas of prevention (79%) and nutrition (77%), confirming that respondents consider these to be of paramount importance. The rate of attitudes towards substance use was high (71%), reflecting the importance of avoiding substances that are harmful to health. Regular physical activity (67%) and emotional balance (66%) scored moderately high, indicating that the majority of young people consider exercise and maintaining mental stability to be important. In contrast, the lowest average scores were in the dimensions of subjective health status (54%), smoking (55%) and telephone and internet use (55%). This is consistent with the observation that adolescents do not consider their own health status to be very good and that their attitudes towards smoking and the use of digital devices are not yet very health-conscious. Regarding alcohol consumption, 58% of respondents had a favourable attitude. The aggression result was 71%, suggesting that the majority reject aggressive behaviour. After exploring attitudes towards health behaviour, we were also interested in determining where health and other fundamental values stand in adolescents’ value systems.

**Figure 2:**
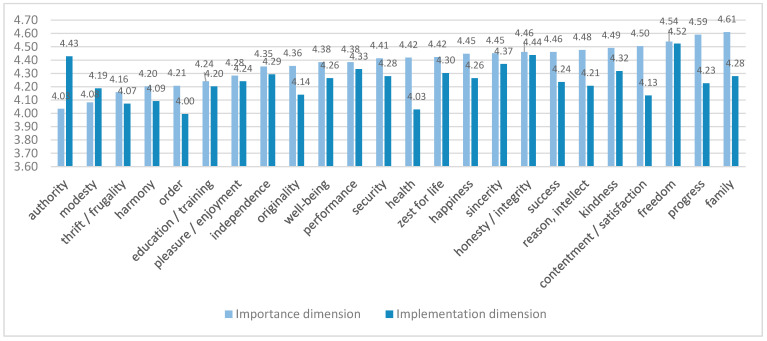
Adolescents’ value preferences (IRVS importance dimension, N = 208, average).

In terms of importance, health (4.03), family (4.28), security (4.28), happiness (4.26), honesty (4.44), and life satisfaction (4.30) are particularly important. The highest average score was achieved by freedom (4.52), followed by honesty (4.44), authority (4.43) and sincerity (4.37). Lower average scores were recorded in the categories of order (4.00), meaning (4.07), and harmony (4.09). The results clearly show that values representing emotional and existential security are most important to the age group surveyed, whereas more abstract, spiritual values are less important. The data are shown in Figure 2.

As shown in Figure 2, we examined not only the importance of values but also their application in everyday life. According to the results, adolescents emphasised family, freedom and success at the practical level as well. In terms of the implementation dimension of values, the most important values were family (4.61), freedom (4.54), an adequate standard of living (4.50), progress (4.59) and success (4.46). Well-being (4.35), kindness (4.49) and performance (4.48) also received high average scores. The lowest scores were related to the categories of modesty (4.08) and heritage (4.03).

These findings suggest that values related to personal development and social security occupy an important place in adolescents’ value systems.

In addition to general values, we considered it important to examine how adolescents assess their own health status. The average score for adolescents’ self-assessed health status is 75.45 on a scale of 0–100, which is moderately good but indicates that increased attention is needed in the area of well-being. These findings describe adolescents’ health-related values, perceived health status, and psychosocial characteristics within the study sample.

In our study, we analysed adolescents’ leisure activities and time-spending habits. On the basis of our results, watching series is the most common pastime among young people, as one in four (22.7%) of the adolescents surveyed indicated this activity as their most frequent pastime. This figure clearly shows the widespread use of streaming platforms among this age group. After watching a series, the most popular activities were playing sports (20.2%) and spending time with friends (19.4%). In addition, many participants read (14.6%), danced (16.0%) or played online games (14.9%). Creative and artistic activities, on the other hand, were hardly mentioned. Only 2.2% like to draw, 0.9% like to paint, and a mere 0.6% like to programme. Musical hobbies (such as playing the piano, guitar, or making music) are practically negligible (0.1–0.4%). Some respondents also mentioned unique activities, such as fishing, pottery, learning Japanese, baking, furniture and candle making, hairdressing, 3D printing, and motorcycling. These activities were mentioned by only 0.1–0.3% of the respondents. The detailed distribution of leisure activities is presented in Table 1.

Based on the phone settings, we calculated the adolescents’ average daily screen time to be 4.5 h. More than 42% of respondents used smartphones most often, while laptops or desktop computers were the second most popular choice (18.9%). Smartwatches (13.9%) and tablets (13.2%) are also quite common. The use of Smart TVs is the lowest (11.4%), while photography was reported by a small proportion of participants (0.7%). In addition to leisure activities, it is important to review which digital devices adolescents prefer for everyday use.

The results suggest that adolescents primarily consume media via digital platforms and devices. The most frequently reported form of media use was smartphones (N = 194, 20.5%), followed by internet use (N = 185, 19.6%) and computer use (N = 174, 18.4%). Podcasts (N = 131, 13.8%) and television viewing (N = 121, 12.8%) were also commonly reported. By contrast, traditional media forms, such as radio listening (N = 85, 9.0%) and reading newspapers (N = 56, 5.9%), were mentioned less frequently. Because participants could select multiple response options, these findings reflect the relative frequency of different media channels in adolescents’ everyday lives rather than their exclusive media preferences. The detailed distribution of media use is presented in Table 2.

Our research revealed that adolescents’ digital presence primarily focuses on video- and image-sharing platforms. The most frequently used application was YouTube, with 157 adolescents (75.5%) reporting its use, followed by Instagram (141 adolescents, 67.8%) and TikTok (137 adolescents, 65.9%). Facebook was used by 97 respondents (46.6%). Among communication platforms, Snapchat was used by 80 adolescents (38.5%), while Messenger, Viber, and WhatsApp were used by 71 adolescents (34.1%), and Discord by 45 adolescents (21.6%). Pinterest was used by 22 respondents (10.6%), while only 13 adolescents (6.3%) mentioned Spotify. Only three respondents (1.4%) indicated that they did not use any of the listed applications. Since participants could select multiple applications, the percentages reflect the proportion of respondents who reported using each application. The distribution of the most frequently used applications is presented in Table 3.

**Figure 3:**
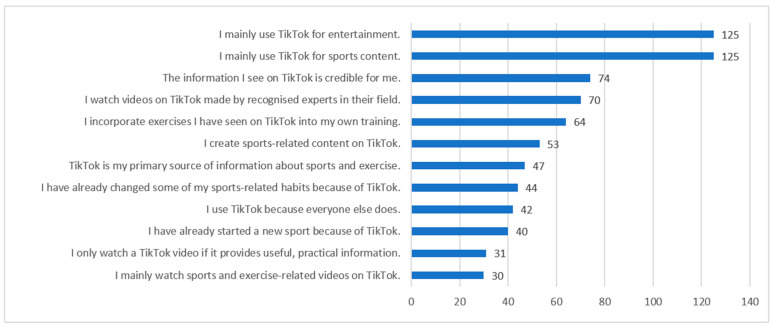
Main reasons for adolescents’ use of TikTok and their sports-related habits (N = 208).

The responses of teenagers revealed that the impact of social media is divided, as most respondents, 110 people (52.9%), said that they are not influenced by the content they see. Moreover, 60 respondents (28.8%) said that social media influences their clothing choices, and 53 respondents (25.5%) said that it influences their self-confidence. The respondents believed that social media content influences their eating habits (27 people, 13.0%) and sporting habits (26 people, 12.5%). On the basis of the responses, the most common motivations for using TikTok are entertainment (125 respondents) and viewing sports-related content (125 respondents). Some respondents had already changed their sports habits as a result of TikTok (44 people), and 53 people had even created sports- and training-related content for the platform themselves. In addition to watching sports and training videos, 30 people specifically search for this type of content, whereas 42 people primarily use TikTok because it is popular among their friends. In terms of usefulness, 31 respondents indicated that they watch videos only when they are practical and informative, whereas 70 respondents specifically follow videos from more reputable professionals. Regarding the platform’s credibility, 74 respondents reported that the information they see on TikTok is reliable. In addition, 40 people took up a new sport as a result of TikTok, and 64 people stated they incorporated TikTok exercises into their own workouts. A total of 47 people indicated that TikTok is their primary source of information on sports and exercise. Our results are shown in Figure 3 below.

Our respondents’ short-form video consumption habits (Reels) show a varied picture.

Most spend less than 1 h a day watching such content (45.2%, 94 people), whereas 36.1% (75 people) spend 1–2 h a day. Fewer respondents reported watching short videos for 3–4 h a day (5.8%, 12 people) or more than 4 h a day (1.4%, 3 people). A total of 11.5% of the respondents (24 people) did not consume this type of content at all. The internet usage of those surveyed covers several areas. These distributions are summarised in Table 4.

The most common activities included communication, chatting and using forums (14.6%, 167 people), obtaining information (14.2%, 163 people) and watching funny short videos (14.2%, 163 people). In addition, learning (14.4%, 165 people), gaming (11.4%, 130 people) and downloading (11.0%, 126 people) also appeared at high rates. Browsing social media sites was reported by 8.4% of respondents (96 people), whereas blogging (1.9%, 22 people) and content creation (2.9%, 33 people) were reported in smaller proportions. Online shopping accounted for 6.7% (77 people) of the total. 3 people (0.3%) reported not using the internet. On the basis of these data, the internet is primarily a place for socialising, obtaining information and entertainment among adolescents. The majority of the respondents had relatively new smartphones; 75.5% (157 people) of the adolescents responded that their smartphone was 0–3 years old, 20.7% (43 people) used a phone that was 3.5–5 years old, and 1.9% (4 people) had a device that was more than 5 years old. A total of 1.9% (4 people) reported not having a smartphone.

Our results show that digital device use and social media play prominent roles in adolescents’ everyday lives and significantly affect their leisure activities, health behaviour and psychological well-being. The average daily screen time of the respondents (4.5 h) is consistent with international data, which shows that digital content consumption among adolescents has steadily increased in recent years [26,27]. Our results also confirm that online platforms, especially TikTok and Instagram, serve not only as entertainment but also as sources of information and lifestyle guidance, as evidenced by changes in sports and eating habits. The data obtained in the dimensions of self-esteem and body image suggest that although the majority of adolescents do not have clinical-level body image disorders, moderate dissatisfaction and uncertain self-esteem are present in the sample. This finding is consistent with previous research, which suggests that the internalisation of unrealistic body ideals seen in social media may increase the risk of body image disorders and self-esteem problems [11]. Our research also shows that values particularly important to adolescents, such as family, freedom, and health, are closely related to their life satisfaction and psychological well-being. In contrast, elevated anxiety levels indicate that psychological support for young people with chronic dermatological conditions is essential as part of complex care. In our work, we first examined whether greater dermatology-related quality of life impairment was associated with poorer general quality of life among adolescents with chronic dermatological diseases. Analysis of the EQ-5D-5L and DLQI scores revealed a significant negative correlation between dermatology-related quality of life and general quality of life (Spearman’s ρ = −0.331, *p* < 0.001). This finding suggest that adolescents reporting greater impairment in dermatology-related quality of life also reported poorer overall quality of life.

We examined whether there was a difference in self-esteem levels among adolescents with chronic dermatological diseases. An analysis using the Rosenberg Self-Esteem Scale (RSES) revealed a significant difference (Mann–Whitney U = 1339,5, Z = −0.0852, *p* < 0.05, two-tailed), indicating that adolescents reporting greater dermatological impairment also reported poorer overall quality of life. We investigated whether greater daily screen time and social media use are significantly associated with lower self-esteem, body image and health behaviour. Digital self-presentation, i.e., self-image building on social media, is closely related to adolescents’ psychological well-being and mental health indicators, which supports the idea that social media use shapes self-esteem and body image in complex ways [13].

In this study, we also analysed the correlations among quality of life, self-assessment, body image, and psychological well-being among adolescents living with chronic dermatological diseases. The EQ-5D-5L quality of life index score was significantly negatively correlated with the DLQI score, indicating that poorer dermatological quality of life was associated with lower overall quality of life. Self-esteem (RSES) also showed a significant negative correlation with DLQI, suggesting that greater skin complaint burden was associated with lower self-esteem. Regarding body image indicators, the self-esteem scale (RSES) was positively correlated with the body image scale, suggesting that a more negative body image was associated with lower self-esteem. A positive correlation was found between life satisfaction (SWLS-H) and self-esteem, suggesting that adolescents with higher self-esteem are more satisfied with their lives.

There are also strong, significant correlations between anxiety (STAI-Y2) and quality of life indicators. Anxiety was negatively correlated with quality of life, self-esteem, and life satisfaction. In contrast, it was closely and positively correlated with the BAT (and BSQ (rs = 0.146, *p* = 0.049) scores, which measure negative body image. Dermatological quality of life (DLQI) was negatively correlated with the EQ-5D-5L quality of life index; i.e., higher DLQI scores (poorer dermatological quality of life) were associated with lower overall quality of life. In addition, the dermatological quality of life scale (DLQI) score was positively correlated with anxiety score (STAI-Y2), indicating that skin diseases represent a greater psychological burden.

Self-esteem (RSES) was closely related to several psychological variables: it was negatively correlated with anxiety and body image, and positively correlated with life satisfaction (SWLS-H) and overall quality of life.

A strong positive correlation was found between the body image scales (BAT and BSQ), confirming the good convergent validity of the two measurement tools. A more negative body image was significantly associated with greater anxiety and lower quality of life. Life satisfaction (SWLS-H) was strongly positively correlated with self-esteem and negatively correlated with anxiety and body image. These findings suggest that greater life satisfaction is associated with lower anxiety and a more positive body image. Lower EQ-5D-5L quality-of-life scores were associated with poorer body image, lower life satisfaction, and greater anxiety. Our findings are therefore consistent with observations that the media has become an increasingly powerful factor in shaping perceptions of chronic illness and attitudes towards health in recent decades, although few studies have examined the consequences of these effects in detail [11]. The detailed correlation coefficients and significance levels are presented in Table 5.

## 4. Discussion

This study shows that the daily lives of adolescents living with chronic dermatological conditions are strongly shaped by digital media. Streaming and social networking are central leisure activities, while creative pursuits are comparatively rare. In this context, quality of life related to skin disease aligned closely with mental health and self-perception: poorer quality of life specific to dermatology clustered with higher anxiety, lower self-esteem and a less favourable body image. Conversely, higher self-esteem and life satisfaction were associated with a more positive perception of health and body image. Taken together, these patterns suggest a mutually reinforcing triad in adolescence—skin burden, digital milieu and psychosocial well-being—in which heightened dermatological impact and intensive online exposure may be associated with greater appearance-focused comparisons and anxiety, while stronger self-related resources (e.g., self-esteem and self-worth) were associated with more favourable psychosocial outcomes.

### 4.1. Comparing Our Findings with Prior Psychodermatological Research

Our findings are also consistent with those of studies that have focused specifically on paediatric psoriasis. Caroppo et al. reported that children with even relatively mild psoriasis experience a measurable reduction in quality of life, and that greater impairment is associated with disease severity and lesion localisation. Our observations support these findings, showing that dermatology-related quality of life is closely associated with adolescents’ psychological well-being, self-esteem, and overall quality of life, regardless of the specific dermatological diagnosis [28]. However, our findings extend previous research by additionally examining adolescents’ media use and health-related values. Studies on digital self-presentation and social comparison have also reported associations between heavy social media use and lower body satisfaction and poorer well-being among young people. Our data are consistent with this literature, showing that negative body image is associated with higher anxiety and lower quality of life [29]. At the same time, not all adolescents reported a strong influence of the media on their attitudes or behaviour. This aligns with research highlighting heterogeneity in susceptibility, moderated by factors such as internalised values, self-regulation, family climate and media literacy. Importantly, our results also add to the existing evidence that ‘screen time’ is always harmful. Although high consumption is associated with less favourable psychosocial indicators overall, a significant proportion of adolescents report using platforms for information, sports, and skills. Other studies have linked these uses to motivation and health-promoting behaviours [30]. This mixed picture suggests the need to move beyond volume-based metrics towards the quality, goals and credibility of the content consumed, alongside individual vulnerability and resilience factors.

### 4.2. Strengths and Limitations

The study’s strengths include a sufficiently powered clinical sample, the use of multiple validated instruments spanning dermatology-specific and generic quality of life, anxiety, self-esteem, body image, health behaviours and personal values, and the inclusion of a study-specific exploratory media questionnaire that captured platform-specific media habits and perceived influence. Limitations temper inference. First, the cross-sectional design precludes causal conclusions (e.g., whether anxiety worsens skin-related quality of life or vice versa). Second, recruitment from a single clinical centre may limit generalisability to community samples and to adolescents without diagnosed skin conditions. Third, although measurement relied on validated tools, some constructs (e.g., media use motives, perceived credibility) are self-reported and subject to recall and social-desirability biases. Fourth, rare diagnoses were represented by small cells, reducing power for between-condition contrasts. Fifth, we did not include objective clinical severity indices, which could clarify the pathways linking disease activity to psychosocial outcomes. Because of the low number of participants in some diagnostic categories, disease-specific subgroup analyses were not feasible. Finally, multiple correlation tests raise the chance of Type I error; future work should pre-register analytic plans and consider correction strategies or multivariate modelling. In addition, the study-specific media questionnaire was developed for exploratory purposes and has not undergone formal psychometric validation; therefore, findings related to these variables should be interpreted with appropriate caution.

### 4.3. Implications for Practice, Policy and Research

The results support integrating brief psychosocial screening (e.g., anxiety, self-esteem, and body image) into routine adolescent dermatology care. Low-threshold referral to psychodermatology or school-based counselling should be considered when appropriate. Given the importance of social media platforms, media literacy and credibility checks (e.g., distinguishing influencer content from evidence-based advice) may be beneficial when incorporated into therapeutic education. Ideally, this would involve parents in strengthening adherence and reducing harmful experimentation. In terms of public health and schools, interventions that combine health promotion focused on appearance-neutral areas such as sleep, activity, and nutrition with value-based education and self-esteem building could mitigate the impact of appearance-focused comparisons. Co-designed digital campaigns involving credible professionals and youth ambassadors could encourage the adoption of reliable skincare behaviours.

In terms of research, longitudinal and mixed-methods designs are required to map temporal directions (e.g., flare-anxiety vs. anxiety-perceived burden) and to test moderation by internalised health values, family climate and media literacy skills. Incorporating objective disease-severity metrics and digital trace data (where ethical) would refine mechanism testing. Finally, intervention trials combining dermatological care with brief psychological skills and media literacy modules could determine whether these integrated approaches improve clinical and quality-of-life outcomes. The adolescent dermatological experience is not an isolated phenomenon; it is intertwined with a high-exposure, comparison-rich media environment and core self-evaluations. A targeted approach that links dermatology, psychology, and digital health education is well positioned to improve quality of life and resilience in this population.

## 5. Conclusions

Among this group of adolescents with chronic dermatological conditions, those with greater impairment in dermatology-related quality of life were found to have poorer psychosocial outcomes. Based on these results, greater dermatology-related quality of life impairment was associated with poorer quality of life, lower self-esteem, more negative body image and higher anxiety. These variables were significantly associated with each other and may jointly contribute to adolescents’ psychosocial well-being. One limitation of the study is its cross-sectional design, which limits the ability to establish clear causal relationships. According to our research data, there is a clear need to consider psychosocial factors in dermatological care. The quality of life of adolescents with skin diseases does not depend solely on the severity of their clinical symptoms but is also closely related to their psychological state, media use and perceptions of health as a value. Based on our results, the majority of respondents paid close attention to influencers’ opinions on dermatological diseases. Similar correlations were confirmed in a study of patients with atopic dermatitis, which found that social media is a key source of information for skin care decisions but that credibility concerns pose a serious dilemma for patients. Our findings suggest that health promotion programmes that incorporate psychosocial support and media literacy could be beneficial. The research also highlights the importance of an interdisciplinary approach: an interdisciplinary approach may contribute to improved dermatological care. For decision-makers, this indicates that the physical and mental health of adolescents cannot be separated. School health promotion and prevention programs should emphasise a critical approach to media use, the strengthening of self-esteem, and the development of awareness of health as a value. In the future, programmes that address symptoms and comprehensively support adolescents’ psychological well-being are needed. In the long term, this approach not only improves individuals’ quality of life but may contribute to at the social and economic levels. Importantly, our study shows only a slice of the whole picture, so it is not possible to determine exactly what the underlying causes are. Regardless, our research shows that psychological factors must be taken into account when treating skin diseases. The quality of life of adolescents living with chronic dermatological diseases was associated not only with the severity of their physical symptoms but also on their mental state, their use of social media, and how they perceive the value of health.

## Figures and Tables

**Table 1:** Distribution of adolescents by leisure activities and hobbies (N = 208).

	N	Percent
Hanging out with friends	152	22.7%
Playing sports	135	20.2%
Playing online games	130	19.4%
Watching TV series	107	16.0%
Reading	98	14.6%
Drawing	15	2.2%
Painting	6	0.9%
Dancing	4	0,6%
Photography	5	0.7%
Programming	3	0.4%
Playing the piano	3	0.4%
Playing the guitar	2	0.3%
Crocheting	2	0.3%
Candle making	2	0.3%
Making music	1	0.1%
Pottery	1	0.1%
Cosplay	1	0.1%
Learning Japanese	1	0.1%
Tinkering	1	0.1%
Baking	1	0.1%
Collecting pebbles	1	0.1%
3D printing	1	0.1%
Hairdressing	1	0.1%
Motorcycling	1	0.1%
		100.0%

**Table 2:** Frequency of digital device and media platform use among adolescents (N = 208; respondents could select more than one option).

	N	Percent
listening to the radio	85	9.0%
watching TV	121	12.8%
using a computer	174	18.4%
using a smartphone	194	20.5%
reading newspapers	56	5.9%
using the internet	185	19.6%
Podcast	131	13.8%
		100%

**Table 3:** Most frequently used applications among adolescents (N = 208; multiple responses allowed).

Application	N	% of Adolescents
YouTube	157	75.5
Instagram	141	67.8
TikTok	137	65.9
Facebook	97	46.6
Snapchat	80	38.5
Messenger/Viber/WhatsApp	71	34.1
Discord	45	21.6
Pinterest	22	10.6
Spotify	13	6.3
None	3	1.4

Note. Multiple responses were allowed; therefore, percentages do not sum to 100%.

**Table 4:** Distribution of adolescents according to daily short-form video viewing time (N = 208).

	Frequency	Percent
Less than 1 h per day	94	45.2
1–2 h per day	75	36.1
I don’t watch short videos	24	11.5
3–4 h per day	12	5.8
More than 4 h per day	3	1.4
Total	208	100.0

**Table 5:** Significant correlations between psychological and quality-of-life variables among adolescents with chronic dermatological diseases (N = 208).

Variable 1	Variable 2	Direction *	Spearman’s ρ (rs)	*p*-Value *
Dermatology Life Quality Index (DLQI)	General quality of life (EQ-5D-5L)	−	−0.331	<0.001
Dermatology Life Quality Index (DLQI)	Rosenberg Self-Esteem Scale (RSES)	−	−0.180	0.009
Dermatology Life Quality Index (DLQI)	State-Trait Anxiety Inventory (STAI-Y2)	+	0.279	<0.001
Rosenberg Self-Esteem Scale (RSES)	Body Attitudes Test (BAT) (rs = 0.255)	+	0.255	<0.001
Rosenberg Self-Esteem Scale (RSES)	Body Shape Questionnaire (BSQ-14) (rs = 0.259)	+	0.259	<0.001
Rosenberg Self-Esteem Scale (RSES)	Body Attitudes Test (BAT)	−	−0.216	0.001
Rosenberg Self-Esteem Scale (RSES)	Body Shape Questionnaire (BSQ-14)	−	−0.117	0.046
Rosenberg Self-Esteem Scale (RSES)	Satisfaction With Life Scale—Hungarian version (SWLS-H)	+	0.249	<0.001
Rosenberg Self-Esteem Scale (RSES)	General quality of life (EQ-5D-5L)	+	0.123	0.038
Rosenberg Self-Esteem Scale (RSES)	State-Trait Anxiety Inventory (STAI-Y2)	−	−0.283	<0.001
Rosenberg Self-Esteem Scale (RSES)	State-Trait Anxiety Inventory (STAI-Y2)	−	−0.182	0.014
Body Attitudes Test (BAT)	Body Shape Questionnaire (BSQ-14)	+	0.513	<0.001
Body Attitudes Test (BAT)	State-Trait Anxiety Inventory (STAI-Y2)	+	0.355	<0.001
Body Shape Questionnaire (BSQ-14)	State-Trait Anxiety Inventory (STAI-Y2)	+	0.264	<0.001
Body Attitudes Test (BAT)	General quality of life (EQ-5D-5L)	−	−0.122	0.040
Satisfaction With Life Scale—Hungarian version (SWLS-H)	General quality of life (EQ-5D-5L)	+	0.312	<0.001
Satisfaction With Life Scale—Hungarian version (SWLS-H)	State-Trait Anxiety Inventory (STAI-Y2)	+	0.239	0.001
Satisfaction With Life Scale—Hungarian version (SWLS-H)	Body Attitudes Test (BAT)	−	−0.209	0.001
Satisfaction With Life Scale—Hungarian version (SWLS-H)	Body Shape Questionnaire (BSQ-14)	−	−0.117	0.046
State-Trait Anxiety Inventory (STAI-Y2)	General quality of life (EQ-5D-5L)	−	−0.252	0.001

* All correlations are Spearman’s rho (two-tailed, *p* < 0.05 considered significant). Positive (+) values indicate favourable associations; negative (−) values indicate inverse relationships where higher scores on one variable correspond to poorer outcomes on the other.

## Data Availability

The datasets generated and/or analysed during the current study are available from the corresponding author upon reasonable request.

## Abbreviations

EQ-5D-5L	EuroQol Five-Dimension Five-Level Questionnaire
ELTE	Eötvös Loránd University
EU	European Union
IPAQ	International Physical Activity Questionnaire
IRVS	Individual Value System Scale
OCEAN	Openness, Conscientiousness, Extraversion, Agreeableness, Neuroticism (personality model)
SWLS-H	Satisfaction with Life Scale—Hungarian version
WHO	World Health Organization
WHO WBI-5	World Health Organization Well-Being Index (5-item version)
